# 9.1 GENOMICS AND PSYCHIATRIC DIAGNOSIS

**DOI:** 10.1093/schbul/sby014.029

**Published:** 2018-04-01

**Authors:** Michael Owen

**Affiliations:** Cardiff University

## Abstract

**Background:**

Recent genomic studies have begun to reveal the genetic architecture of psychiatric disorder and to give important insights into the relationship between the psychiatric syndromes that form the basis of current taxonomy. These studies have demonstrated the highly polygenic nature of psychiatric disorders, and have indicated that many individual genetic associations are shared across multiple disorders in a way that points to extensive biological pleiotropy and challenges the biological validity of existing diagnostic approaches.

**Methods:**

I will present genomic data, predominantly from the study of rare variants, that support the idea of a neurodevelopmental continuum, in which schizophrenia and bipolar disorder, together with childhood neurodevelopmental disorders, such as ID, ASD and ADHD represent the diverse range of outcomes that follow from disrupted or deviant brain development and furthermore that, within the neurodevelopmental continuum, severe mental illnesses occupy a gradient of decreasing neurodevelopmental impairment as follows: ID, ASD, schizophrenia and bipolar disorder. I will also present findings indicating that common genetic variation modifies the outcome of neurodevelopmental impairment explaining in part the diversity of psychiatric outcomes. Finally, I will explore how genetic data might be used to inform novel approaches to patient stratification which will be informative for prognosis and treatment response and facilitate the identification of novel drug targets.

**Results:**

Finally, despite the undoubted complexity and the fact that much of the genetic risk remains unaccounted for at the DNA level, there are encouraging signs that the genes implicated in schizophrenia converge onto sets of plausible biological processes. In particular, the data point to synaptic function and histone modification and implicate mechanisms involved in brain plasticity that are important in development and in learning and cognition. While these are almost certainly not the only processes involved, they provide robust entry points for clinical and basic neuroscience research.

**Discussion:**

N/A

